# Remarks

**Published:** 1842-07-16

**Authors:** 


[*Remarks.*—The difficulty in the diagnosis of this case was, to decide whether the cerebral symptoms were dependant upon an actual inflammation of the brain, or were purely secondary to the suppression of urine and the disease of the kidneys. The pathological changes, and the whole history of the case, induce us to adopt the former opinion,—that the brain symptoms were not merely produced by the alteration of the blood from renal disease. In order of time, however, the cerebral lesions probably followed the inflammatory alteration of the kidneys, and were, perhaps, caused by it. A renal disease will occasionally give rise to positive inflammation, as well as to mere sympathetic disturbance of the brain. The treatment is in these cases not a little modified.

Where the history of the case is unknown, as in the present instance, the discrimination between the two forms of cerebral affection will be almost impracticable; but where the disease is traced from the beginning, the symptoms connected with inflammation will in general be found to be more intense than those of pure functional disorder. If the brain be not the seat of actual inflammation, the patient usually becomes dull and loses his memory, but very rarely falls into violent agitation or delirium.—W. W. G.]

